# Deciphering transcriptional dynamics of cardiac hypertrophy and failure in a chamber-specific manner

**DOI:** 10.17305/bb.2023.8997

**Published:** 2023-12-01

**Authors:** Dan Zhang, Jianming Liu, Haiying Xiao, Jun Li, Ling Cao, Guang Li

**Affiliations:** 1 Key Laboratory of Medical Electrophysiology, Ministry of Education and Medical Electrophysiological Key Laboratory of Sichuan Province, and Collaborative Innovation Center for Prevention of Cardiovascular Diseases, Institute of Cardiovascular Research, Southwest Medical University, Luzhou, China; 2 Department of Nephrology, Affiliated Hospital of Southwest Medical University, Southwest Medical University, Luzhou, China

**Keywords:** Cardiac hypertrophy (CH), heart failure (HF), transcriptome, dynamic changes, pathogenesis

## Abstract

Pressure overload-induced pathological cardiac hypertrophy (CH) is a complexed and adaptive remodeling of the heart, predominantly involving an increase in cardiomyocyte size and thickening of ventricular walls. Over time, these changes can lead to heart failure (HF). However, the individual and communal biological mechanisms of both processes remain poorly understood. This study aimed to identify key genes and signaling pathways associated with CH and HF following transverse aortic constriction (TAC) at four weeks and six weeks, respectively, and to investigate potential underlying molecular mechanisms in this dynamic transition from CH to HF at the whole cardiac transcriptome level. Initially, a total of 363, 482, and 264 differentially expressed genes (DEGs) for CH, and 317, 305, and 416 DEGs for HF were identified in the left atrium (LA), left ventricle (LV), and right ventricle (RV), respectively. These identified DEGs could serve as biomarkers for the two conditions in different heart chambers. Additionally, two communal DEGs, elastin (*ELN*) and hemoglobin beta chain-beta S variant (*HBB*-*BS*), were found in all chambers, with 35 communal DEGs in the LA and LV and 15 communal DEGs in the LV and RV in both CH and HF. Functional enrichment analysis of these genes emphasized the crucial roles of the extracellular matrix and sarcolemma in CH and HF. Lastly, three groups of hub genes, including the lysyl oxidase (*LOX*) family, fibroblast growth factors (*FGF*) family, and NADH-ubiquinone oxidoreductase (*NDUF*) family, were determined to be essential genes of dynamic changes from CH to HF.

## Introduction

Cardiovascular diseases are the leading cause of death worldwide and are associated with immense health and emotional and economic burdens [1]. Heart failure (HF) is the end stage of various cardiac diseases that severely affects patients’ quality of life, and the factors leading to HF are numerous and complex. Pathological myocardial remodeling is the key pathophysiological process causing HF. Cardiac hypertrophy (CH) is considered a fundamental and potentially indispensable step in the myocardial response to pressure overload and one of the main features of pathological myocardial remodeling. Whereas pathological hypertrophy is initially an adaptive response to stimuli, pathological hypertrophy can lead to HF and is regulated by several cellular signaling pathways [2]. Hypertrophy of terminally differentiated cardiomyocytes maintains or even improves contractile functions, but its persistent state can lead to cardiomyocyte death, fibrosis, and eventually HF, highlighting the urgent need for effective therapeutic strategies that target underlying regulators and signaling pathways. CH is considered a common precursor and one of the main reasons for HF [2–5]. Several mechanisms, mediators, and signaling pathways play an important role in the cardiac adaptation to the transcriptional regulatory network that facilitates cardiac adaptation and the transition from hypertrophy to overt HF. Previous studies have provided an overview of some negative regulators [6] on CH and discussed a part of differentially expressed genes (DEGs) [7], signaling pathways, and molecular mechanisms associated with pathological and physiological CH based on specific regions within the heart [3, 8–10]. With the aging of the population, the incidence of HF is increasing, but treatment options for this patient group are limited [11]. The conventional goal for treating CH is to relieve clinical symptoms and improve cardiac function, delay disease progression, and prevent or slow the onset of HF and sudden death. With the deepening of the pathophysiological mechanism of CH, treatment has transformed into molecularly targeted drugs and gene therapy [9, 12–14]. Although there has been considerable progress in medicine, the pathogenesis of CH is still not fully understood. Moreover, the molecular mechanisms underlying the transition from adaptive hypertrophy to myocardial decompensation have not been fully elucidated. Genetic testing can identify asymptomatic CH patients before the onset of clinical disease and thus guide the diagnosis, treatment, and prognosis of the disease. Therefore, more efficient and accurate diagnostic techniques are urgently needed. It is worth noting that the key molecules that promote or inhibit the dynamic switch from CH to HF have not yet been systematically studied and elaborated.

In this study, we developed a pressure overload mouse model using the transverse aortic constriction (TAC) strategy to systematically analyze and elaborate the gene expression patterns in different chambers of the whole heart. By analyzing the transcriptomic data of samples from different compartments, we can reveal more molecular markers involved in the two types of disease and identify the critical factors responsible for the transition from CH (four weeks post-TAC) to HF (six weeks post-TAC). Our results will help to shed new light on the pathogenesis of CH and HF and provide additional material and a research base for subsequent studies on CH and HF.

## Materials and methods

### Transverse aortic constriction (TAC) surgery and echocardiographic recording in mouse models

Male C57BL/6J mice (eight weeks old, 23–25 g, *n* ═ 27) were anesthetized by intraperitoneal administration of 1% pentobarbital (0.005 mL/g body weight). TAC surgery was performed as previously described [15, 16]. The Vevo 3100 system (FUJIFILM Visual Sonics Inc) was used to assess cardiac function and record echocardiographic parameters, including left ventricular end-diastolic diameter (LVIDd), left ventricular end-systolic diameter (LVIDs), left ventricular end-diastolic posterior wall thickness (LVPWd), short-axis shortening rate (FS), and ejection fraction (EF) of mice anesthetized with the aerosol anesthetic isoflurane. Control groups (Sham four weeks and six weeks) underwent the same operation without TAC (*n* ═ 3 per group).

### RNA extraction, library preparation, and transcriptome sequencing

According to the manufacturer’s instructions, total RNA was extracted from the samples using the RNeasy Mini Kit (Qiagen GmbH, Hilden, Germany). We collected 27 RNA samples of all conditions from nine mice, including three CH mice, three HF mice, and three Sham mice. RNA purity, concentration, and integrity were checked and measured using the Bioanalyzer 2100 system (Agilent Technologies, CA, USA). Sequencing libraries were prepared from 3 µg of high-quality RNA per sample according to the manufacturer’s recommendations. After clustering of the index-coded samples, library preparations were sequenced on an Illumina Hiseq platform and 150 bp paired-end reads were generated.

### Quality control and reads mapping

We filtered raw data in “fastq” format using in-house Perl scripts and Trimmomatic v. 0.35 software [17]. After removing reads with adapters, ploy-N, and low-quality reads, we obtained the clean data and simultaneously calculated its Q20, Q30, and GC content. The reference genome and gene model annotation files were directly downloaded from the genome website (version: GRCm38, https://www.ncbi.nlm.nih.gov/assembly/GCF/_000001635.20/). We aligned the clean RNA sequencing (RNA-seq) data to the GRCm38 reference genome using Hisat2 v2.0.5 [18]. Subsequently, we used Picard tools v.1.92 (http://broadinstitute.github.io/picard) to assign read group information which includes library, lane, and sample identity and to remove duplication caused by PCR.

### Quantification of gene expression levels

In our analyses, a gene or isoform was considered ubiquitously expressed if its Fragments Per Kilobase of transcript sequence per Millions base pairs (FPKM) was greater than 0.1 across all samples. To identify DEGs between different conditions, the value of one was added to each FPKM value before the log2 transformation. Gene expression levels in each sample were quantified as read counts generated with featureCounts v1.5.0-p3 [19]. Subsequently, FPKM of each gene was calculated based on gene length and the reads count mapped to this gene. The DESeq2 R package (version 1.16.1) was utilized to analyze differential gene expression was analyzed [20, 21].

### Correlation between samples and principal component analysis (PCA)

Correlation coefficients were calculated based on the FPKM values of all genes in each sample to visually represent sample differences between groups and duplication within groups. In this study, we required *R^2^* (Pearson correlation coefficient) between biological replicate samples to be at least greater than 0.7. A sample correlation heat map was drawn according to the correlation coefficient between samples. PCA analysis was performed for FPKM using the SMARTPCA program in the EIGENSOFT package v. 5.0.2 [22], and eigenvectors were obtained from the invariant matrix using the R function “reigen.” The programming language R displayed the correlation analysis and PCA analysis graphs.

### Identification and clustering of differentially expressed genes (DEGs)

Differential expression analysis between any two comparisons was performed using the DESeq2 R package v. 1.16.1 [21]. The resulting *P* values were adjusted using Benjamini and Hochberg’s approach to control the false discovery rate. Genes with an absolute value of log2FC greater than one and an adjusted *P* value less than 0.05 as determined by DESeq2 were assigned as differentially expressed. After normalizing the rows of expression data, we used hierarchical clustering to perform cluster analysis on the gene expression values of the DEGs. We created a heat map of clustering and a Venn diagram using R language. Through the Venn diagram, it is possible to identify communal or individual differential genes for a particular comparison combination.

**Figure 1.**
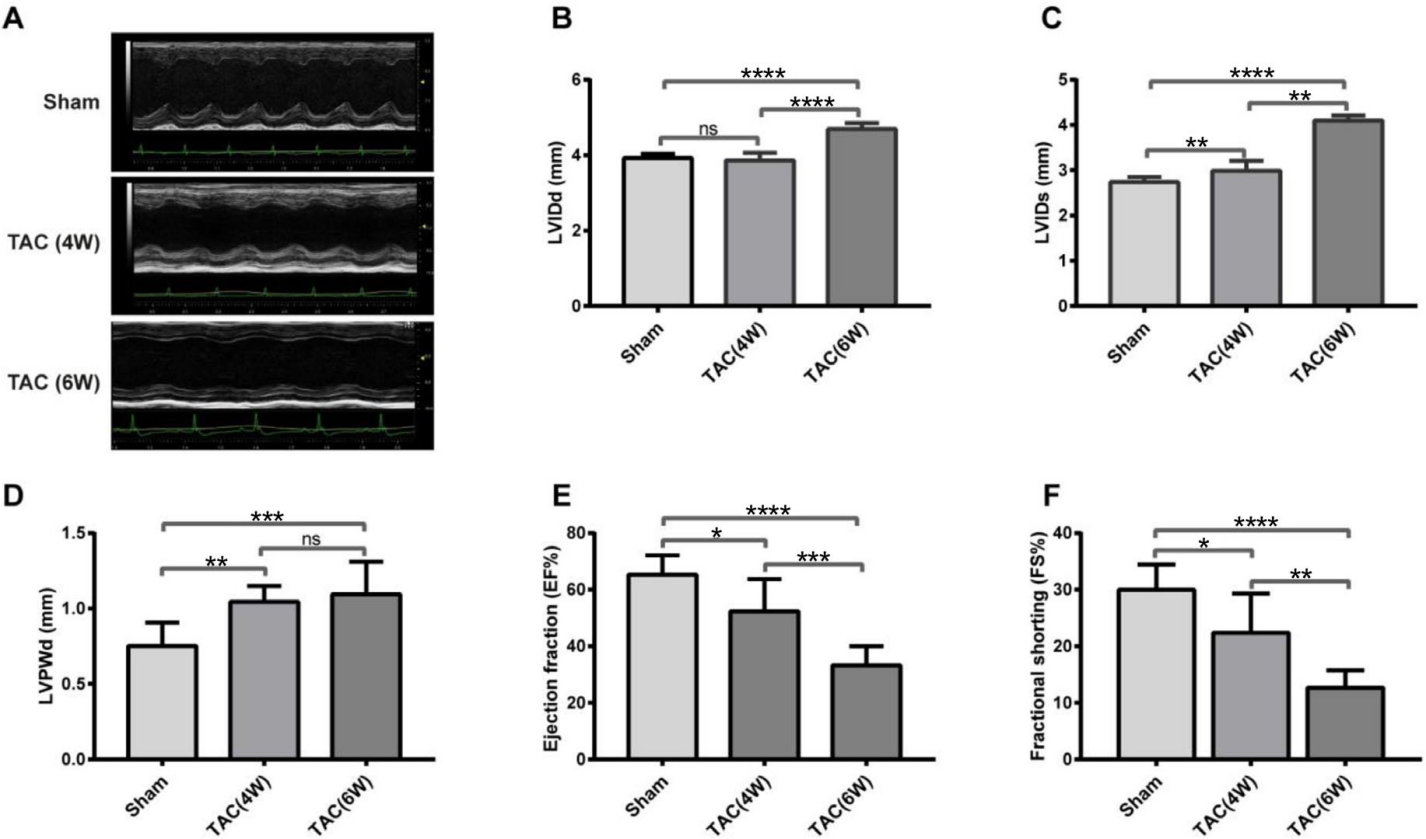
**Cardiac function of TAC model mice by echocardiogram**. (A) Representative M-mode echocardiographic imaging of Sham and TAC mice four and six weeks (4W and 6W) post-TAC; (B) Left ventricular end-diastolic diameter (LVIDd); (C) Left ventricular end-systolic diameter (LVIDs); (D) Left ventricular posterior wall end-diastolic thickness (LVPWd); (E) Left ventricular ejection fraction (EF%); (F) Left ventricular fractional shorting (FS%). TAC: Transverse aortic constriction.

### Enrichment analysis of DEGs

To analyze the screened DEGs at the functional level, we performed Gene Ontology (GO) enrichment analysis and Kyoto Encyclopedia of Genes and Genomes (KEGG) pathway analysis using the “clusterProfiler” package in R language [23]. A corrected *P* value less than 0.05 was considered significantly enriched by DEGs. To further investigate various reactions and biological pathways, we conducted a Reactome analysis using the “ReactomePA” package in R. An adjusted *P* value less than 0.05 was used as the threshold of significant enrichment in the Reactome pathway [24].

### Protein–protein interaction (PPI) network analysis

PPI analysis of DEGs was conducted using the STRING database (http://string-db.org/), which predicts PPIs. An interaction with a combined score greater than 0.4 was considered statistically significant. Subsequently, the PPI networks were visualized using Cytoscape v.3.6.1 [25]. The Molecular Complex Detection (MCODE; version 1.4.2) Cytoscape plugin allows the clustering of a given network based on topology to identify densely connected regions.

### Ethical statement

The protocol was approved by the Ethics Committee for Animal Experiments at Southwest Medical University (approval No. 20180309024) and adhered to the US National Institutes of Health Guidelines for the Care and Use of Laboratory Animals.

### Statistical analysis

All data were analyzed using R statistical software (The R Project for Statistical Computing) and *GraphPad* Prism 7 (La Jolla, CA) and presented as mean ± standard deviation (SD). The Shapiro–Wilk test was used for the normal distribution assay and Pearson correlation was utilized for correlation analysis. Statistical analysis was performed using one-way ANOVA followed by Tukey’s method or unpaired two-tailed Student’s *t*-tests. A *P* value less than 0.05 was considered to indicate a significant difference. The Benjamini–Hochberg procedure was used to control the false discovery rate.

## Results

### Decreased cardiac function in TAC mice

We performed 2-D-guided M-mode echocardiography on mice four and six weeks post-TAC and Sham surgery (Figure 1A). LVIDd was significantly increased in six weeks (4.69 ±0.158 mm) post-TAC (Figure 1B) compared to four weeks (3.86 ± 0.201 mm) and Sham (3.92 ± 0.115 mm), with no observed significant difference between four weeks post-TAC and Sham mice. LVIDs have been progressively increased from four (2.988 ± 0.221 mm) to six weeks (4.096 ± 0.114 mm) post-TAC relative to Sham mice (2.74 ± 0.111 mm) (Figure 1C). At four weeks, LVPWd significantly increased compared to Sham (0.752 ± 0.155 mm), however, no significant difference was observed in LVPWd at four (1.045 ± 0.104 mm) and six weeks (1.094 ± 0.217 mm), indicating that left ventricle (LV) hypertrophy had occurred by four weeks post-TAC (Figure 1D). TAC mice showed a significant decline in cardiac function over time post-TAC surgery. Both EF (52.30 ± 11.38%) and FS (22.39 ± 6.012%) slightly but significantly decreased at four weeks in TAC mice vs Sham (EF═65.32 ± 6.772%; FS═30 ± 4.469%), while they decreased dramatically at six weeks post-TAC, with the values of EF═33.20 ± 6.82% and FS═12.68 ± 3.069%. EF and FS decreased by 19.93% and 25.37% at four weeks and 49.17% and 57.73% at six weeks, respectively (Figure 1E and 1F). The alterations among Sham, CH four weeks post-TAC (TAC4W), and HF six weeks post-TAC (TAC6W) indicated the development of cardiac decompensation from the compensatory stage (Figure 1).

### Sequencing data statistics

To construct a comprehensive transcriptomic heart map of the mouse following TAC, we generated an RNA-seq data set using 27 samples isolated from eight weeks old C57BL/6J male mice. Approximately 127.2 million paired-end raw reads were generated on average, yielding 125.5 billion clean reads and 188.3 G base pairs for the study after quality control. Further details of the RNA-seq data are provided in Table S1. Q20 and Q30 of all clean reads were more than 99.2% and 95.8%, respectively, indicating that the RNA-seq data were of high quality for transcriptome analysis.

### Overview of the landscape of mouse entire heart transcriptome

With all the biological samples combined, a raw data matrix of FPKM values was generated. This consisted of 54,533 genes annotated in the Ensembl GRCm38 mouse reference genome across 27 samples. Most of the reads were in genic regions as shown in Figure S1A. After removing zero values, adding one and applying a log2 transformation to the FPKM values, a final data matrix of 34,793 genes was generated for subsequent analyses. The distribution of gene expression levels across different samples using the final FPKM matrix is illustrated in Figure S1C. Both correlation analysis and PCA were performed on all models based on their FPKM values. The results showed that Pearson correlation coefficients (*R^2^*) were all greater than 0.7 in the correlation analysis, and samples were clustered according to their source in the PCA analysis, as seen in Figure S1B. These results demonstrated that three different heart chambers exhibited unique gene-expression profiles.

### Identifying DEGs and enrichment analysis in different chambers in cardiac hypertrophy (CH) and heart failure (HF)

**Figure 2.**
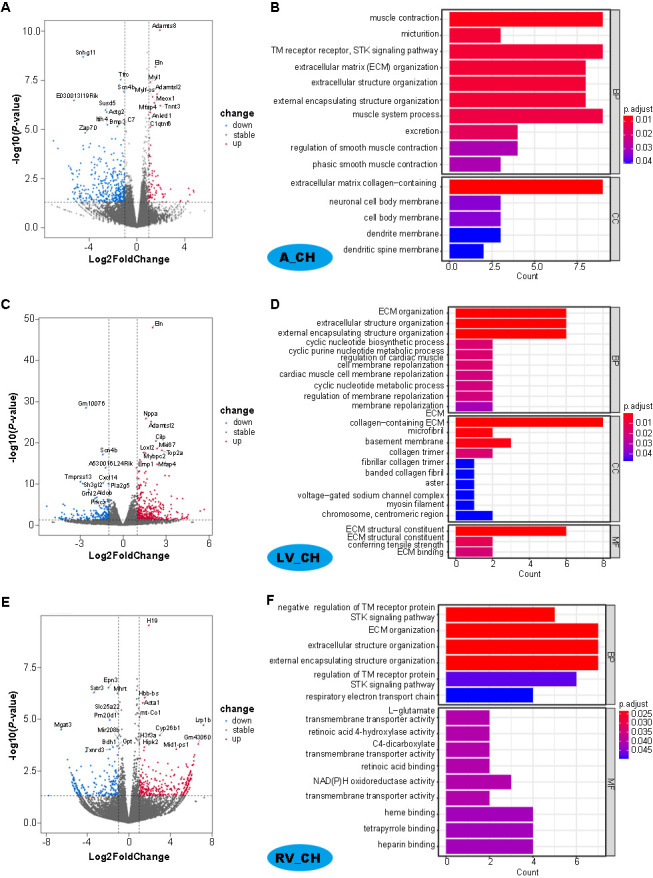
**Analysis of differentially expressed genes (DEGs) in the same position under the CH-Sham**. Volcano plots of DEGs for in LA (A), LV (B), and RV (C), blue dots represent down-regulated DEGs; red dots represent up-regulated DEGs. The most significant 10 DEGs gene names are shown. The top 10 GO terms in biological processes, cellular components, and molecular functions for DEGs in LA, LV, and RV are shown in the right part of the picture (B and D). CH: Cardiac hypertrophy; LA: Left atrium; LV: Left ventricle; RV: Right ventricle.

The DESeq2 software was used to identify DEGs in the CH and HF in left atrium (LA), LV, and right ventricle (RV), respectively, compared to Sham corresponding to the same regions of heart. For the CH group, 363 DEGs, including 68 up-regulated and 295 down-regulated DEGs, were identified in the LA using criteria of *P* value less than 0.05 and the absolute value of log2FC greater than one. Similarly, in the LV, 482 DEGs, including 332 up-regulated and 150 down-regulated DEGs, and in the RV 264 DEGs, including 165 up-regulated and 99 down-regulated DEGs, were identified, respectively (Figure 2). The most significant DEGs in CH are associated with maintaining myocardial fibers, cardiac structure and functions, cardiac conduction, and energy synthesis and metabolism. Conversely, for the HF group, using the same identification criteria as the CH group, we identified 317 DEGs (85 up-regulated and 232 down-regulated) in LA; 305 DEGs (171 up-regulated and 134 down-regulated) in the LV; and 416 DEGs (254 up-regulated and 162 down-regulated) in the RV (Figure S2). These genes are closely related to cardiac remodeling, cardiac rhythm, cardiac contraction, energy, and oxygen delivery, and several HF marker genes were detected, suggesting these genes play an essential role in HF. The top 10 up-/down-regulated DEGs (Figure 2 and Figure S2) may serve as potential biomarkers of CH and HF in different heart chambers. The top 10 DEGs for each chamber in CH and HF are also listed in Table 1.

**Table 1:** Top 10 up-/down-regulated DEGs in LA, LV, and RV

	**HF**		**CH**	
	**UP**	**DOWN**	**UP**	**DOWN**
**LA**	*SPON2*	*TEF*	*ADAMTS8*	*SNHG11*
	*ADAM19*	*GM9824*	*ELN*	*TFRC*
	*GM40841*	*PER2*	*MYL1*	*SCN4B*
	*NPAS2*	*PER3*	*ADAMTSL2*	*E030013I19RIK*
	*GM47644*	*GM45819*	*MYLF-PS*	*SUSD5*
	*5430431A17RIK*	*ANGPTL7*	*MEOX1*	*ACTG2*
	*KRT19*	*ENTPD4B*	*TNNT3*	*C7*
	*IL18R1*	*GPR22*	*MFAP4*	*ITIH4*
	*ALAS2*	*WEE1*	*ANKRD1*	*BMP3*
	*HBB-BT*	*COQ10B*	*C1QTNF6*	*ZAP70*
**LV**	*YPEL2*	*INMT*	*ELN*	*GM10076*
	*ADAM19*	*COQ10B*	*NPPA*	*SCN4B*
	*ELN*	*AGT*	*ADAMTSL2*	*A530016L24RIK*
	*CCR5*	*CYP2B10*	*CILP*	*TMPRSS13*
	*5430431A17RIK*	*TCAP*	*MKI67*	*CXCL14*
	*ARNTL*	*ADH1*	*TOP2A*	*SH3GL2*
	*MYH7B*	*TMPRSS13*	*LOXL2*	*PLA2G5*
	*ADAMTSL2*	*CCBE1*	*MYBPC2*	*GRHL2*
	*SPON2*	*ANO5*	*EMP1*	*ALDOB*
	*ADCY7*	*WDFY1*	*MFAP4*	*PRKCZ*
**RV**	*CYP26B1*	*RPL36*	*H19*	*EPN3*
	*BCL3*	*EIF3M*	*HBB-BS*	*SSTR3*
	*RRAD*	*FAM174B*	*ACTA1*	*MHRT*
	*DIO2*	*ZFP949*	*MT-CO1*	*SLC25A22*
	*GCK*	*FBXL22*	*LRP1B*	*PM20D1*
	*NGEF*	*GPR22*	*CYP26B1*	*MGAT3*
	*MAPT*	*G0S2*	*H3F3A*	*MIR208B*
	*MSLN*	*TNP2*	*GM43060*	*GPT*
	*ADAMTSL2*	*HOPX*	*HIPK2*	*BDH1*
	*ACTA1*	*WIF1*	*MID1-PS1*	*TXNRD3*

HF: Heart failure; CH: Cardiac hypertrophy; LA: Left atrium; LV: Left ventricle; RV: Right ventricle.

### Comparison of enriched GO functions and pathways in CH vs HF

To explore the biological processes, cellular components, and molecular functions associated with DEGs, we performed GO analysis for the DEGs in the two different pathological conditions (right parts of Figure 2 and Figure S2). Moreover, GO enrichment of DEGs can aid in further classification and enhance understanding of their functions in different diseases. For CH, the most significant GO terms associated with DEGs in the LA and LV were biological processes and cellular components except in the case of the RV. Three GO terms related to extracellular matrix (ECM), structure, and encapsulating organization were shared across all three chambers. In contrast, muscle contraction, muscle system process, and collagen-containing ECM were shared in LA and LV. Other GO terms were specific to each chamber: GO terms related to smooth muscle contraction were identified in the LA, while GO terms associated with cardiac muscle cell membrane repolarization, myosin filament, and sodium channel complex were found in the LV. GO terms linked to energy metabolism and transmembrane transporter activity of a substance were observed in the RV (Figure 2 and Figure S2). However, for the HF group, the top enriched GO items in the LA were mainly associated with circadian rhythm, substances involved in reduction–oxidation reaction (REDOX) processes, nerve conduction, and oxygen/gas transport. Apart from the five GO terms related to ECM structure and composition shared in the LV and RV, the most significant GO terms in the LV were related to amino acid transmembrane transport, regulation of cardiac/striated muscle cell apoptotic process, fat cell differentiation, and chemokine/cytokine/immune receptor activity. Finally, GO terms in the RV were associated with muscle/myotube/striated muscle cell differentiation, myocardial structure development and maintenance, including sarcomere, myofibrils, and contractile fibers (Figure S2). Detailed information on the GO terms in CH and HF from the three individual chambers are shown in Table S2.

**Figure 3.**
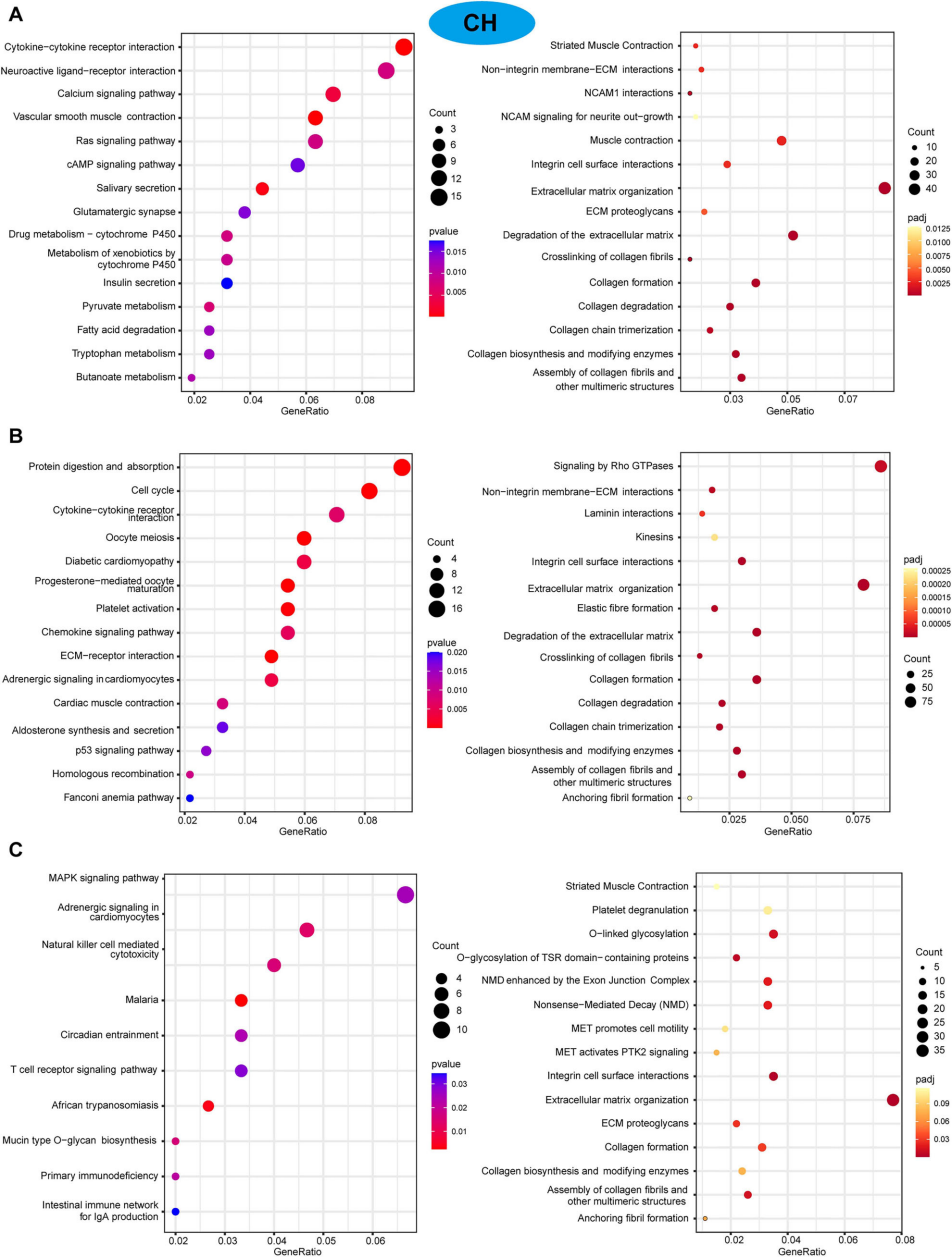
**Statistics of pathway enrichment for DEGs from the LA, LV, and RV under the CH condition in mice**. The KEGG enrichment pathway is on the left, and the Reactome enrichment pathway is on the right of the picture. (A) Enrichment pathway of the DEGs in the LA; (B) Enrichment pathway of the DEGs in the LV; (C) Enrichment pathway of the DEGs in the RV. The *Y*-axis represents the pathway entry, and the *X*-axis indicates the gene ratio of DEGs corresponding to the pathway entry. The size of the point represents the number of DEGs. DEGs: Differentially expressed genes; LA: Left atrium; LV: Left ventricle; RV: Right ventricle; CH: Cardiac hypertrophy; KEGG: Kyoto Encyclopedia of Genes and Genomes.

To comprehensively investigate biological systems and metabolic pathways, we conducted KEGG and Reactome pathway enrichment analyses for the DEGs in the two conditions. While Reactome emphasizes comparative analysis of biological reactions and physiological processes, KEGG primarily addresses gene function and metabolic pathways. For CH, the most significant KEGG and Reactome pathways in the LA, LV, and RV are shown in Figure 3. The KEGG pathway of the cytokine–cytokine receptor interaction was enriched in both the LA and LV, while adrenergic signaling in cardiomyocytes was shared in the LV and RV. Using Reactome analysis, 10 of the top 15 significant pathways were enriched in the LA and LV, four in the LA and RV, and two in the LV and RV. The top 15 KEGG and Reactome pathways of LA, LV, and RV in HF are shown in Figure S3. Two KEGG pathways were shared across all three heart chambers, one was the same as in CH in the LA and LV, and five were common to the LV and RV. The detailed pathways specific to each chamber are shown in Figure 3 and Figure S3, and also Table S3. Furthermore, two Reactome pathways were shared in the LA and RV, and five were associated with ECM organization and collagens in the LV and RV and five were associated with ECM organization and collagens in the LV and RV (Figure 3 and Figure S3). The detailed KEGG and Reactome pathways of CH and HF in the LA, LV, and RV can be found in Tables S3 and S4, respectively.

### Key genes and alterations of signaling pathways in the progression from CH to HF

**Figure 4.**
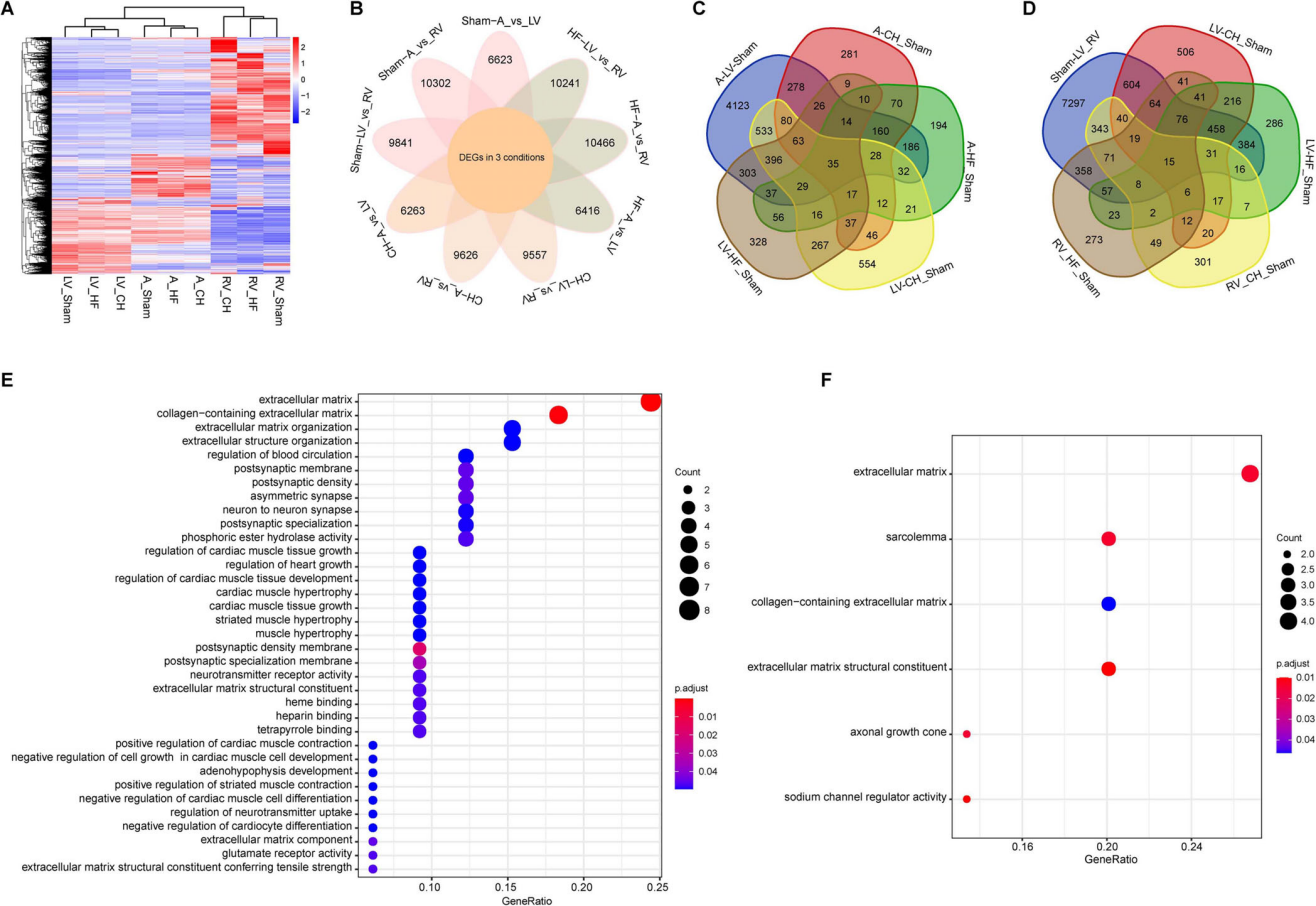
**Key genes and functional changes in the progression from CH to HF.** (A) Hierarchical clustering analysis of DEGs of 27 samples; (B) Venn diagram shows DEGs in three conditions across LA, LV, and RV; (C) 35 DEGs shared in CH and HF from LA and LV samples; (D) 15 DEGs were shared in CH and HF from two ventricular samples of the heart (LV and RV); (E) GO terms (BP, CC, MF) for the common 35 DEGs in the chambers on the left heart (LA and LV) from two pathological conditions; (F) GO terms for the common 15 DEGs in LV and RV from two pathological conditions. CH: Cardiac hypertrophy; HF: Heart failure; DEGs: Differentially expressed genes; LA: Left atrium; LV: Left ventricle; RV: Right ventricle; GO: Gene Ontology; BP: Biological process; CC: Cellular component; MF: Molecular function.

Figures 2 and 3 and Figure S2 and S3 display the similarities and differences in gene expression patterns among the various chambers in CH and HF, respectively. However, there were significant differences in the numbers of DEGs among the LA, LV, and RV in the two different conditions (Figure 4A and 4B). To identify potential key genes in the dynamic progression from normal to CH and eventually to HF, we focused on the DEGs identified in the LA and LV in both, CH vs Sham and HF vs Sham. Across these two conditions, 35 DEGs were detected in both the LA and LV (Figure 4C). Subsequently, we directed our analysis on the 35 significant genes consistently represented across all conditions for GO analysis. Multiple GO terms were closely related to ECM-related components, hypertrophy, cardiac structure/function, and nerve conduction (Figure 4E). Meanwhile, 15 shared DEGs were identified in the LV and RV in CH and HF comparisons (Figure 4D). The most significant GO terms were related to sarcolemma, ECM-related components, axonal growth cone, and sodium channel regulator activity (Figure 4F).

### Constructing PPI network and module analysis of key DEGs in CH and HF

**Figure 5.**
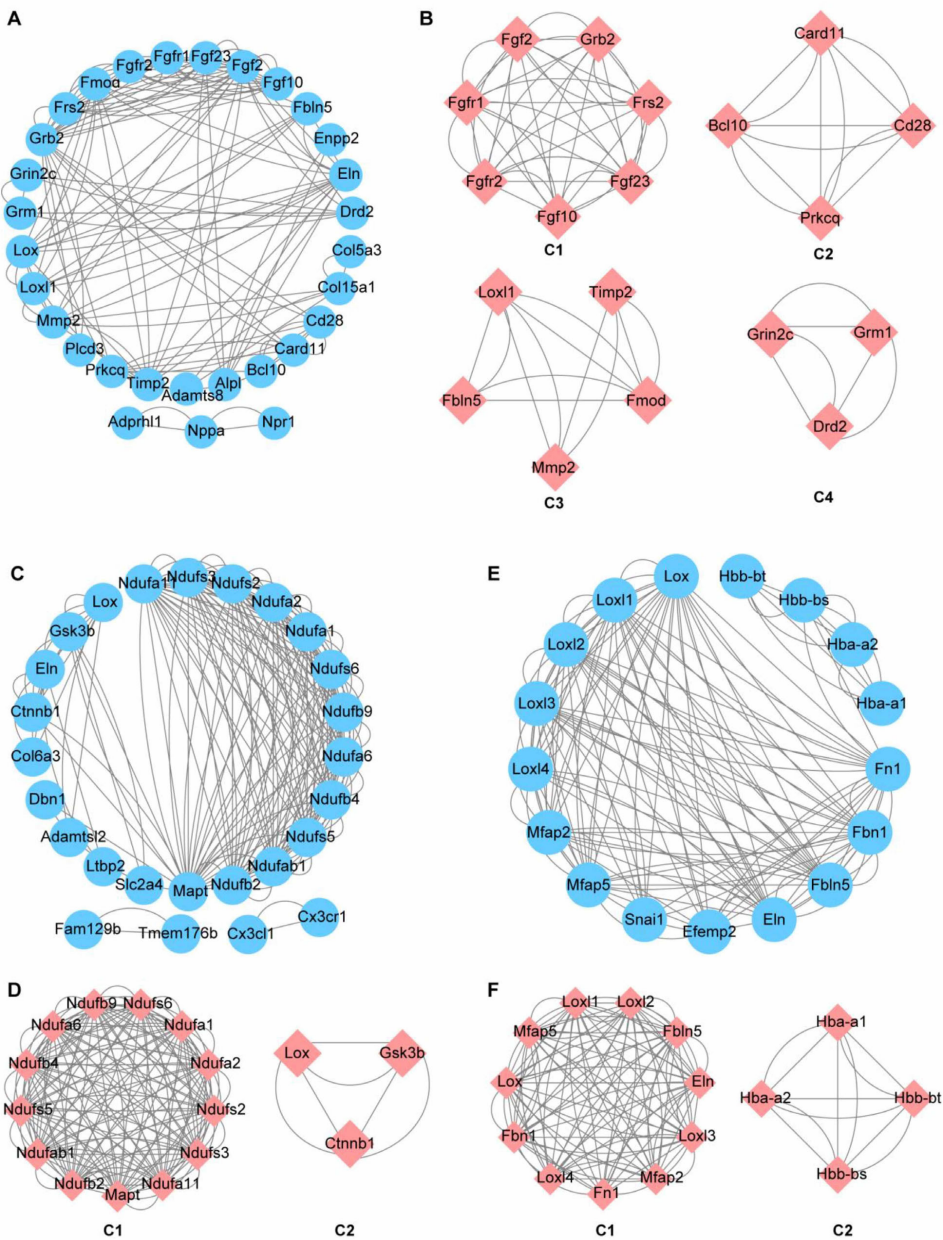
**Constructing PPI network of potential key genes from CH to HF.** (A) The PPI network of 35 DEGs shared in CH and HF conditions in the LA and LV; (B) Four key modules of the PPI network in the LA and LV; (C) The PPI network of 15 DEGs in CH and HF conditions is shared in the LV and RV; (D) Two key modules of the PPI network in the LV and RV; (E) The PPI network of 2 DEGs shared in CH and HF conditions in LA, LV, and RV; (F) Two key modules of the PPI network in three chambers. The modules are performed by the MCODE plugin in Cytoscape software. PPI: Protein–protein interaction; CH: Cardiac hypertrophy; HF: Heart failure; DEGs: Differentially expressed genes; LA: Left atrium; LV: Left ventricle; RV: Right ventricle; MCODE: Molecular complex detection.

**Figure 6.**
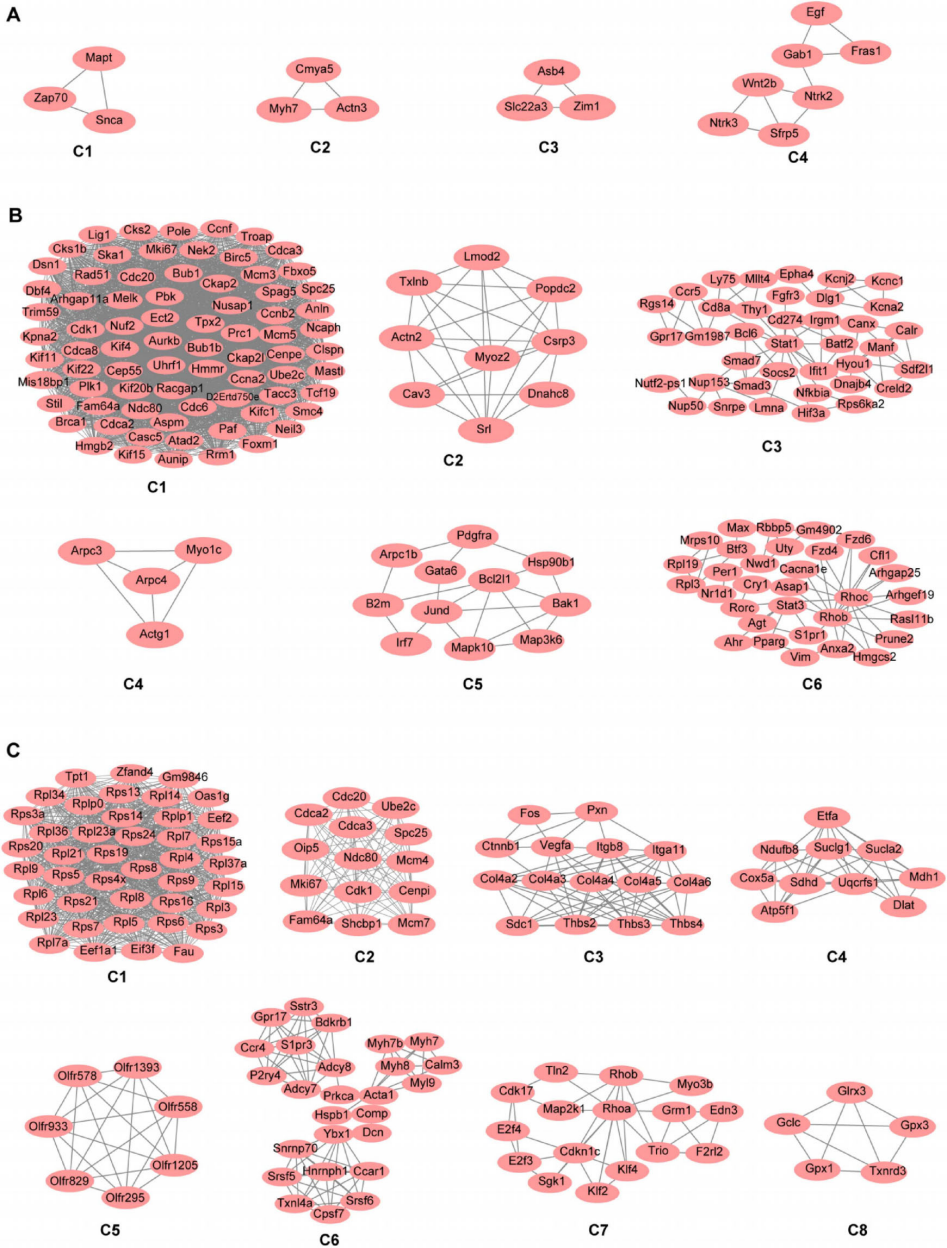
**The key modules derived from constructed PPI networks of potential essential genes from CH to HF.** (A) Four key modules of 140 DEGs are shared in CH and HF conditions in the LA; (B) Six key modules of 102 DEGs in the LV; (C) Eight key modules of 85 DEGs in the RV. The MCODE plugin in Cytoscape software performs the modules. PPI: Protein–protein interaction; CH: Cardiac hypertrophy; HF: Heart failure; DEGs: Differentially expressed genes; LA: Left atrium; LV: Left ventricle; RV: Right ventricle; MCODE: Molecular complex detection.

To further explore the key molecules or proteins involved in the progress of CH toward HF, we identified co-expression networks of DEGs both in CH and HF. The PPI networks were constructed in the LA–LV combination and LV–RV combination, and three chambers in both CH and HF, respectively. This approach could increase our understanding of the complex molecular mechanisms of HF. Sub-network modules obtained from three PPI networks were demonstrated in Figure 5. With a confidence score cutoff of 0.4, the final network contained 30 nodes and 128 edges in the LA–LV (Figure 5A), and we identified four function modules, shown in red in Figure 5B. Similarly, 26 nodes and 186 edges in the LV–RV, with two function modules (Figure 5C and 5D) and 17 nodes and 138 edges across all chambers, with two function modules presented. (Figure 5E and 5F). We identified four, six, and eight sub-modules using DEGs shared in CH and HF in the LA, LV, and RV, respectively (Figure 6). The large and complex PPI networks constructed using shared DEGs in CH and HF are shown in Figure S4.

## Discussion

In this study, we conducted a comparison between CH and HF at the whole heart level, taking into account the different chambers. This approach addresses the integrative physiological effects of TAC at both the CH and HF stage. We outlined the transcriptome profiles of the whole heart under CH and HF conditions. Compared with the Sham group, we identified hundreds of unique DEGs in each of the three chambers under these two pathological conditions. Only two DEGs, elastin (*ELN*) and hemoglobin beta chain-beta S variant (*HBB-BS*), were shared across the whole heart, suggesting a change in mechanisms during the development of HF after TAC. Elastin is an ECM protein with a unique biochemical structure that provides elasticity to organs and tissues, including the heart and blood vessels. Both genetic and acquired cardiovascular diseases are associated with elastin and elastic fibers, which inhibit smooth muscle cell proliferation and promote actin filament bundles’ organization. Elastin-derived peptides, or elastokines, stimulate the migration and proliferation of monocytes and fibroblasts [26, 27]. In the heart, the abnormal accumulation of a large number of fibroblasts is accompanied by severe fibrosis, which leads to impaired cardiac function. The other commonly shared DEG, the *HBB-BS* gene, encodes a beta polypeptide chain found in adult hemoglobin, which transports oxygen to various peripheral tissues. It is associated with glucose/energy metabolism, suggesting changes of oxygen delivery and energy in CH and HF. These results preliminarily indicate that *ELN* and *HBB-BSS* could be potential clinical biomarkers or therapeutic targets for CH and HF. However, further studies are urgently needed to confirm these findings. In addition, we performed a separate analysis of the expression of nine potential indicator genes associated with cardiac structure/function and fibrosis, including *ELN*, *HBB-BS*, *MYH*6, *MYH*7, *NPPA*, *NPPB*, *COL1A1*, *COL1A2*, and *COL3A1*. Their expressions were significantly higher at four and six weeks compared to the Sham group, suggesting that CH and remodeling had already occurred by the fourth week (Figure S5A). The fibrosis-related genes *COL1A1*, *COL1A2*, and *COL3A1* showed even higher expression in the CH group than in the HF group in LA and LV. These results are associated with extensive ECM remodeling in the LA and LV, suggesting that the fibrosis-related genes may dynamically change in the mediation of HF (Figure S5C). Heat maps were constructed using the genes reported to be associated with CH and HF in our dataset (Figure S5B). The results showed that the expression of these genes exhibited distinctive changes in the LA, LV, and RV.

To gain a comprehensive understanding of the pathogenesis of CH and HF, we compared the gene expression patterns at the chamber levels. This study identified 140 (LA), 102 (LV), and 85 (RV) common DEGs between the CH vs Sham and HF vs Sham at entire heart levels, which indicates that these shared DEGs play a crucial role in the pathogenesis of HF in the whole heart and exhibit similar damage effects in individual chambers. Although the top 10 DEGs in the two different states were rarely identical across different compartments, most of their GO items were similar, mainly related to the ECM, muscle system processes/contraction, and cardiac conduction. Notably, fat cell differentiation, muscle cell differentiation/development, apoptosis of striated muscle cells, and cytokine binding were specific to HF, indicating that the structural remodeling of the heart is more intensified, and the inflammation is more severe than in the CH.

In the LA, the pyruvate, butanoate, drug, xenobiotics metabolism, and fatty acid degradation pathways were specific to CH. This indicates a shift in atrial metabolism and an accumulation of metabolic small molecule products in CH, but not in HF.

In the LV, which is the primary area affected by TAC, several pathways are shared between CH and HF. These include chemokine, cytokine, adrenergic, aldosterone related, platelet activation KEGG pathways, as well as ECM, collagens, laminin, and integrin-related Reactome pathways. The results suggest that a greater number of inflammatory pathways are involved in the occurrence and progression from CH to HF, and that cardiac structure and conduction pathways are altered. Moreover, the involvement of ECM and collagen fiber-related pathways in both states suggests that fibrosis is developing in the myocardium of the LV, even though myocardial hypertrophy may serve as a compensatory mechanism to maintain the integrity of the function and structure of the damaged heart. Fibrosis occurs due to the excessive deposition of many ECM proteins in myocardial tissue caused by the long term increase of pressure load. This pathological remodeling process of myocardial interstitial expansion will eventually increase myocardial stiffness, resulting in final HF. Notably, activation of both the sympathetic nervous system and the renin-angiotensin–aldosterone system is sufficient to cause cardiomyocyte death [28–30]. However, more KEGG pathways were specific to CH and HF. The unique KEGG pathway in CH indicates that changes in the p53 pathway accompanied by the activation of the RhoA/Rho kinase signaling pathway, may trigger compensatory myocardial hypertrophy of the LV thereby maintaining cardiac structure and contraction. Whereas the specific pathways of HF mainly focus on physiological rhythm and the interaction between muscle and nerve. Importantly, the cGMP-PKG signaling pathway is a crucial pathway that causes HF.

In the RV, the KEGG pathways, such as the MAPK signaling pathway and adrenergic signaling in cardiomyocytes, continue to play crucial roles in CH. Furthermore, an increased number of immune cell-related KEGG pathways were found to be enriched in CH. The cellular components of the immune response, including T cells, B cells, mast cells, natural killer cells, and dendritic cells, are associated with the pathogenesis of CH. Concurrently, the energy metabolism and muscle contraction Reactome pathways suggest that an enhanced myocardial energy metabolism facilitates muscle compensation, maintaining normal function during CH. Contrarily, apart from ECM and muscle contraction, the hormone pathways, insulin secretion, TNF signaling pathway, and O-linked glycosylation were significantly enriched in HF. It is known that overexpression of TNF-α in normal myocardium directly causes myocardial insulin resistance, aggravating cardiac dysfunction and ventricular dilatation following ischemia. Low *PKG* activity increases the resting tension in myocardial cells, leading to myocardial hypertrophy, fibrosis, and vascular stiffness in patients with HF [31, 32]. In TAC-induced HF, decreased NO synthesis results in the obstruction of the NO-SGC-cGMP signaling pathway, further intensifying cardiomyocyte hypertrophy and ventricular remodeling [33].

To identify the key genes and their bio-signature in the progression from CH to HF at the LV, LA, and RV, we first screened 35 overlapping DEGs in two different states in the LA and LV and 15 common DEGs in the LV and RV. Functional analysis revealed that the 35 shared and 15 common DEGs are mainly involved in the ECM, cardiac structure or function, and signal transduction. Following this, we identified the top five genes, including *LOXL2*, *LOXL3*, *FBN1*, *MFAP5*, and *MFAP2* as hub nodes with the most incredible degrees in the MCODE sub-network which had the highest score for the shared 2 DEGs. Similarly, *MAPT*, *NDUFB*2, *NDUFAB*1, *NDUFS*5, and *NDUFB*4 were identified in the LV and RV, while *FGFR1*, *FRS*2, *FGF10*, *FGF2*, and *MMP2* were identified in the LA and LV. Lysyl oxidase (*LOX*) family-associated genes, including *LOX* and *LOXL1*, and the *ELN* gene were significantly enriched in the sub-network modules of the three comparison groups. As mentioned earlier, when the integrity of the elastic fiber is compromised, ECM proteins are produced and remodeled, which can further alter the mechanical behavior of the cardiovascular system [27]. It is becoming increasingly evident that myocardial fibrosis directly contributes to myocardial remodeling and prevents the regeneration of myocardial tissue in the damaged area. LOX is a copper-dependent extracellular enzyme that catalyzes lysine-derived crosslinks in collagen and elastin. The *Fibrillin 1* (*FBN1*) gene encodes a member of the fibrillin family, the encoded protein being a large ECM glycoprotein and a structural component of 10–12 nm calcium-binding microfibrils. MFAP2 and MFAP5 are major antigens of elastin-associated microfibrils and are candidates for involvement in ECM organization and elastic fiber formation.

In cardiovascular disease, the fibroblast growth factors (FGFs) family is implicated in cardiac remodeling through different mechanisms, which can contribute to HF. FGF2 induces CH by activating FGF receptor 1c (FGFR1c) and MAPK signaling. FGF receptor substrate 2α (FRS2α) is the primary mediator of signaling in the FGF pathway. Multiple epidemiological and patient studies have concluded that FGF23 acts directly on the myocardium or via elevations in blood pressure to cause left ventricular hypertrophy [34–36]. The hormonal effects of circulating klotho protein and FGF23 on the vascular and heart have contributed to arterial stiffness and left ventricular hypertrophy. MMPs are zinc-dependent endopeptidases that play an essential role in degrading various collagens. *MMP2* has been identified as a potential biomarker of myocardial fibrosis in patients with hypertrophic cardiomyopathy [37]. NDUFB2, NDUFAB1, NDUFS5, and NDUFB4 proteins have NADH dehydrogenase and oxidoreductase activity. The NADH-ubiquinone oxidoreductase (*NDUF*) gene family transfers electrons from NADH to the respiratory chain. In summary, the critical hub genes we screened have high confidence.

This study is based on a relatively small sample size which warrants discussion. Although our data have demonstrated that CH occurred four weeks post-TAC, and a significant decline in cardiac function and HF occurred at six weeks post-TAC. When measuring cardiac function using echocardiography, it is advisable to include more parameters to obtain comprehensive indicators of both long-axis and short-axis, such as interventricular septal diameter at end-diastole (IVSd) and left anterior descending artery diameter at end-diastole (LADd) as well as heart weight and lung weight measurements. In addition, regarding the expression of hub genes in dynamic changes of CH and HF, this article presents a bioinformatics analysis study that still requires experimental and external verification. Our future work will involve experimental validation of these results and focus on exploring the biological functions and molecular mechanisms.

## Conclusion

In conclusion, our findings suggest that *ELN* and *HBB-BS* might be potential biomarkers in patients with CH and early HF based on the comprehensive analysis conducted across three cardiac chambers. Furthermore, we demonstrated that gene expression during the development of HF is characterized by dynamic sets of overlapping transcripts regulated by specific interrelated mechanisms. Notably, each cardiac chamber exhibits unique gene expression patterns and regulatory mechanisms. Our results showed the cardiac ECM is composed of numerous molecules, including collagen, elastin, fibrin, lox, MMPs, fibronectin, laminin, fibrillin, glycoproteins, and proteoglycans. The ECM is forming a dynamic environment that plays an active and crucial role in regulating cellular events and cardiac remodeling [38, 39]. ECM-associated genes, the *FGFs* family, and respiratory electron transport-associated genes play significant roles in the dynamic progression of HF. These genes along with their associated signaling pathways contribute to the identification of independent DEGs, providing new insights into the distinct mechanisms underlying CH and HF. The common DEGs identified in this study shed light on the potential shared mechanism of CH and HF. Furthermore, the identified hub genes have the potential to serve as biomarkers or therapeutic targets in hypertrophy and early HF.

## Supplemental Data

Supplementary data are available at the following link: https://www.bjbms.org/ojs/index.php/bjbms/article/view/8997/2815.
